# Metformin Does Not Inhibit Exercise-Induced Lipolysis in Adipose Tissue in Young Healthy Lean Men

**DOI:** 10.3389/fphys.2018.00604

**Published:** 2018-05-23

**Authors:** Eva Krauzová, Petr Tůma, Isabelle de Glisezinski, Vladimír Štich, Michaela Šiklová

**Affiliations:** ^1^Department for the Study of Obesity and Diabetes, Third Faculty of Medicine, Charles University, Prague, Czechia; ^2^Second Department of Internal Medicine, University Hospital Královské Vinohrady, Prague, Czechia; ^3^Department of Hygiene, Third Faculty of Medicine, Charles University, Prague, Czechia; ^4^INSERM, UMR1048, Obesity Research Laboratory, Institute of Metabolic and Cardiovascular Diseases, University of Toulouse, Paul Sabatier University, Toulouse, France; ^5^Department of Clinical Biochemistry and Sports Medicine, Toulouse University Hospital, Toulouse, France

**Keywords:** metformin, microdialysis, lipolysis, human adipose tissue, exercise, lactate

## Abstract

**Objective:** Metformin was shown to exert an antilipolytic action in adipose tissue (AT) that might mediate beneficial effects on lipid metabolism in diabetic patients. However, during exercise, the inhibition of induced lipolysis in AT would limit the energy substrate supply for working muscle. Thus, the aim of this study was to investigate whether metformin exerts inhibitory effect on exercise-induced lipolysis in subcutaneous adipose tissue (SCAT) (Moro et al., 2007) in humans.

**Approach:** Ten healthy lean men underwent two exercise sessions consisting of 60 min of cycling on bicycle ergometer combined with (a) orally administered metformin and (b) metformin locally administered into SCAT. Microdialysis was used to assess lipolysis *in situ* in SCAT. Glycerol, metformin and lactate were measured in dialysate and plasma by enzyme colorimetric kits and capillary electrophoresis.

**Results:** Metformin levels increased continuously in plasma during 3 h after oral administration, and peaked after 3.5 h (peak concentration 4 μg/ml). Metformin was detected in dialysate outflowing from SCAT and showed a similar time-course as that in plasma with the peak concentration of 1.3 μg/ml. The lipolytic rate in SCAT (assessed as glycerol release) increased in response to exercise (4.3 ± 0.5-fold vs. basal; *p* = 0.002) and was not suppressed either by local or oral metformin administration. The lactate levels increased in plasma and in dialysate from SCAT after 30–60 min of exercise (3.6-fold vs. basal; *p* = 0.015; 2.75-fold vs. basal; *p* = 0.002, respectively). No effect of metformin on lactate levels in SCAT dialysate or in plasma during exercise was observed.

**Conclusion:** Metformin did not reduce the exercise-induced lipolysis in SCAT. This suggests that metformin administration does not interfere with the lipid mobilization and energy substrate provision during physical activity.

## Introduction

Metformin and physical activity are recommended as the first therapeutic agents to improve glycemic control in prediabetes and type 2 diabetes patients (Ortega et al., 2014). Metformin improves various features of systemic metabolism, such as insulin sensitivity, glycated hemoglobin levels, or plasma cholesterol in patients with type 2 diabetes (Robinson et al., 1998). It has been shown that metformin also reduces plasma concentration of free fatty acids (FFA) and it was hypothesized that this reduction was caused partially by inhibition of lipolysis in adipose tissue (AT) (Flechtner-Mors et al., 1999). In 3T3-L1 cells as well as in human primary adipocytes metformin inhibited catecholamine- and ANP-stimulated lipolysis through activation of AMP-activated protein kinase (AMPK) (Zhang et al., 2009; Bourron et al., 2010). Thus, it could be hypothesized that metformin might inhibit the exercise-induced lipolysis in AT as this is mediated – among other pathways – by catecholamines and ANP. As the inhibitory effect of metformin for both of these pathways was demonstrated either *in vitro* or *in situ* condition with pharmacological doses of metformin, we aimed in this study to verify whether metformin will inhibit lipolysis also in a physiological condition (i.e., exercise).

However, the inhibition of AT lipolysis during physical activity would affect fatty acids mobilization necessary to supply energy for working muscle and heart. In this respect, inhibition of lipolysis by metformin might be considered as undesirable effect of this drug, and thus metformin administration might influence the tolerance of an exercise of long duration. Although metformin was shown to enhance the insulin-sensitizing effect of exercise in insulin resistant patients (Ortega et al., 2014), or to induce higher oxygen consumption and lower lactate response during exercise (Johnson et al., 2008), the effects of metformin on exercise-induced lipolysis in AT has not been investigated, yet. Thus, in this study, we examined the effect of metformin on lipolysis in subcutaneous adipose tissue (SCAT) during an acute bout of exercise using microdialysis technique *in situ* in healthy lean men. It should be mentioned, that this study was carried out in young healthy lean men, as in obese or in diabetic patients the exercise–induced or catecholamine-stimulated lipolysis is impaired (Stich et al., 2000b; Verboven et al., 2016) and the possible antilipolytic effect of metformin could be masked. Thus, we aimed to demonstrate the antilipolytic effect of metformin during exercise in standard physiological condition. Moreover, we have assessed pharmacokinetics of metformin in the dialysate outflowing from SCAT after a single oral administration of metformin. The study could bring important evidence based recommendations to combination of physical activity and metformin therapy.

## Materials and Methods

### Subjects

Ten lean men (age 27.2 ± 0.4 years; BMI 23.6 ± 0.5 kg/m^2^) were recruited for the study. Exclusion criteria were: no weight change within 3 months before the study, smoking, hypertension, impaired fasting glucose, diabetes, hyperlipidemia, drug, or alcohol abuse. This study was carried out in accordance with the recommendations of The Ethical committee of the Third Faculty of Medicine (Charles University, Prague, Czechia). The protocol was approved by the Ethical committee of the Third Faculty of Medicine (Charles University, Czechia). All participants provided written informed consent prior the start of the study.

### Experimental Protocol

**FIGURE 1:**
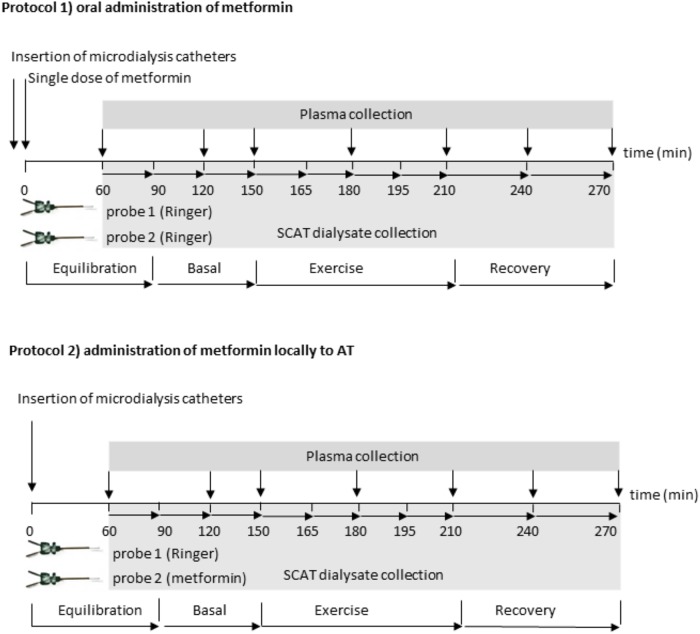
Scheme of the experimental protocols: two probes were connected to a microperfusion pump and perfused at a flow rate 2.5 μl/min. In the Protocol 1: oral metformin administration, both probes were perfused by sterile Ringer’s solution; after 90 min of equilibration, two basal dialysate fractions were collected in 30 min interval, then the subjects performed 60-min exercise bout at 50–55% 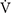
O2peak followed by 60 min of recovery period. Dialysate was collected in 15 min intervals during exercise and in 30 min intervals at the recovery period. Blood samples were taken in 30 or 60 min intervals. Glycerol and lactate was measured in dialysate collected from the first probe, metformin pharmacokinetics was analyzed in the dialysate from the second probe. In the Protocol 2: local metformin administration, the first probe was perfused by sterile Ringer’s solution, the second probe was perfused by Ringer’s solution with 1 mM metformin, (flow rate 2.5 μL/min). After 90 min of equilibration two basal dialysate fractions were collected in 30 min interval, then the subjects performed 60-min exercise bout at 50–55% 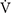
O2peak followed by 60 min of recovery period. Dialysate was collected in 15 min intervals during exercise and in 30 min intervals at the recovery period. Blood samples were taken in 30 or 60 min intervals. Glycerol and lactate were measured in dialysate collected from the both probes.

The subjects underwent complete clinical investigation in the fasted state, including anthropometric and body composition measurement (Bodystat QuadScan 4000; Bodystat Ltd., British Isles). After that the catheter was placed in their antecubital vein, and two microdialysis probes (20 mm × 0.5 mm; 20 kDa cutoff; Carnegie Medicine, Stockholm, Sweden) were inserted percutaneously after epidermal anesthesia (1 mL of 1% Mesocain, Zentiva, Czechia) into the SCAT at a distance of 10 cm from the umbilicus. The probes were connected to microperfusion pump (Harvard Apparatus, France) and perfused at a flow rate 2.5 μl/min. Subjects performed two protocols consisting of a 60 min exercise bout with at least 1 week interval between them, in random order. The exercise intensity corresponding to 55–60% of coronary heart rate reserve calculated according to Karvonen formula (Karvonen et al., 1957) was chosen as it represents optimal intensity for significant increase of lipolysis in healthy lean men (Moro et al., 2007). In protocol 1, metformin (Teva Pharmaceuticals, Czechia) was administered orally (2250 mg/single dose) 2.5 h before the start of the exercise. Both microdialysis probes were perfused with Ringer solution. In the dialysate from one probe glycerol and lactate concentrations were measured. In the dialysate from the second probe metformin concentration was measured. In protocol 2, metformin was administered locally into SCAT by perfusion into one microdialysis probe (1 mM, 2.5 μl/min). The second probe was perfused by Ringer solution (control). Glycerol and lactate concentrations were measured in the dialysate collected from both probes. According to reported pharmacokinetics of metformin in plasma, the exercise bout was subjected at expected peak levels after one-dose metformin administration (FDA; 2.64 ± 0.82 h). The detailed protocol description is depicted in **Figure 1**.

In *in vitro* experiment, the recovery of metformin through microdialysis probe was assessed using zero-flow method as described before (Siklova-Vitkova et al., 2009).

### Plasma Analyses

Plasma levels of glucose, insulin, and lipid parameters were determined using standard methods in certified laboratories. FFA in plasma, and glycerol in plasma and dialysate were measured using enzymatic colorimetric kits (Randox, Crumlin, United Kingdom). Lactate and metformin in dialysate and plasma were analyzed by capillary electrophoresis, as described before (Tuma, 2014).

### Statistical Analysis

Statistical analysis was performed using GraphPad Prism 6 (GraphPad Software, Inc., San Diego, CA, United States). The differences in the concentration of analytes (glycerol, lactate, metformin, FFA) in time during experimental protocols were analyzed by One-way ANOVA with Bonferroni *post hoc* analysis. The difference in concentration of analytes (glycerol, lactate, metformin, FFA) in plasma or dialysate between the Protocol 1 and 2 or between the two probes in Protocol 1 was analyzed using Two-way ANOVA with Bonferroni *post hoc* analysis. Data are presented as mean ± SEM. Differences at the level of *p* < 0.05 were considered statistically significant.

## Results

### *In Vitro* Relative Recovery of Metformin

**FIGURE 2:**
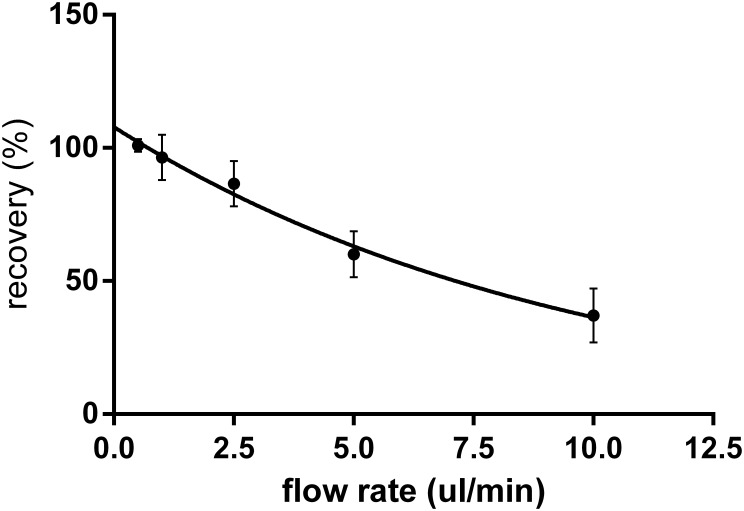
*In vitro* relative recovery (%) of metformin for the microdialysis probes with cut-off 20 kDa in relation to the flow rate of perfusion (*n* = 6). Relative recovery was calculated as the ratio of metformin concentration in dialysate to concentration in the stock solution.

Relative recovery (Bourron et al., 2010), defined as the ratio of “*metformin concentration in dialysate outflowing from the probe*” and “*metformin concentration in stock solution*,” was determined for different flow rates. The relationship between RR and the perfusion flow rates is presented in **Figure 2**. The flow rate of 2.5 μl/min was chosen as the best compromise between the magnitudes of RR and flow rate for all further experiments.

### Pharmacokinetics of Metformin in the Dialysate From Adipose Tissue After Single Oral Dose

**FIGURE 3:**
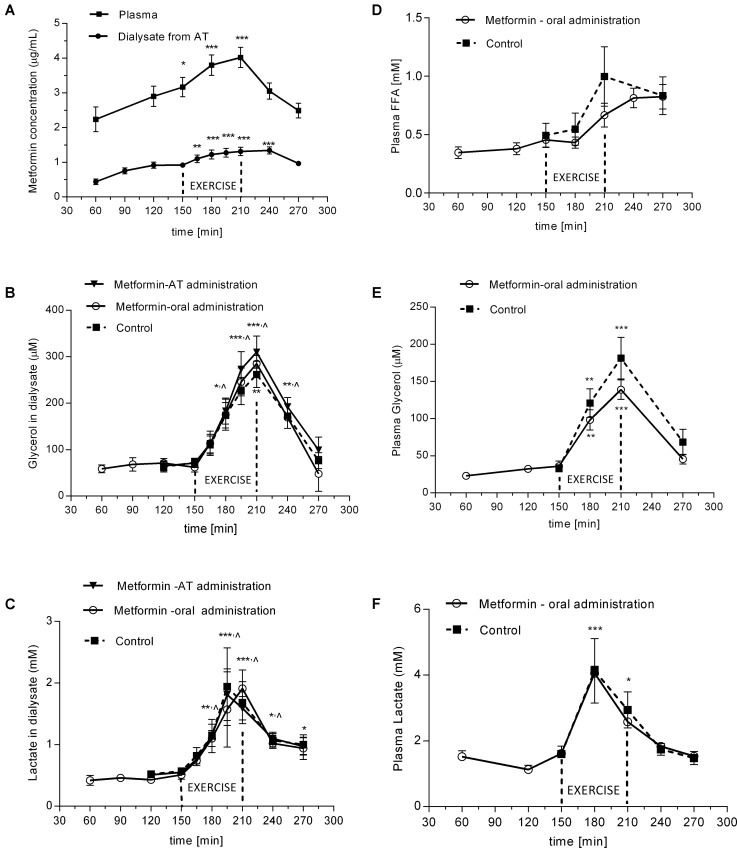
Time-course of metformin, glycerol, FFA, and lactate during experimental exercise protocols with or without metformin treatment: **(A)** metformin levels in plasma and in dialysate outflowing from SCAT after oral administration of metformin; **(B)** glycerol levels in dialysate outflowing from SCAT; **(C)** lactate levels in dialysate outflowing from SCAT; **(D)** FFA levels in plasma; **(E)** glycerol levels in plasma; **(F)** lactate levels in plasma. Values are means ± SEM represented by vertical bars. Significant difference from of pre-exercise levels (baseline): ^∗^*p* < 0.05, ^∗∗^*p* < 0.01, ^∗∗∗^*p* < 0.001 (One-way ANOVA with Bonferroni *post hoc* analysis). **^∧^**significant values apply to all three curves. SCAT, subcutaneous adipose tissue; FFA, free fatty acids.

After administration of a single dose of 2250 mg the metformin levels were steadily increasing in plasma as well as in SCAT dialysate for 3.5 h and declined thereafter (**Figure 3A**). Concentration of metformin in dialysate outflowing from SCAT ranged between 0.44 and 1.34 μg/mL, which represent approximately 30% of the concentration in plasma (**Figure 3A**).

### Effect of Locally and Orally Administered Metformin on Lipolysis in SCAT During Exercise

Ten healthy lean men underwent two experimental protocols with acute bout of exercise and metformin administration: (1) orally (2250 mg/acute dose) 2.5 h before the start of exercise; (2) locally directly into AT by microdialysis probe (1 mM, 2.5 μl/min). Anthropometric and biochemical parameters of the subjects, who underwent the experimental protocol are shown in **Table 1**.

**Table 1:** Anthropometric and biochemical parameters of the subjects.

	healthy lean men (*n* = 10)
Age (years)	27.2 ± 1.4
Weight (kg)	79.1 ± 7.7
BMI (kg/m^2^)	23.6 ± 1.6
Fat mass (%)	11.5 ± 3.6
Fat-free mass (%)	88.5 ± 3.6
Cholesterol (mmol/L)	3.7 ± 0.6
HDL-C (mmol/L)	1.3 ± 0.2
Triglycerides (mmol/L)	0.7 ± 0.1
Glucose (mmol/L)	5.0 ± 0.3
Insulin (mU/L)	3.7 ± 1.8
HOMA-IR	0.8 ± 0.4

*Data are presented as mean ± SD; BMI, body mass index; HOMA-IR, homeostasis model assessment of the insulin resistance index; HDL-C, HDL cholesterol*.

The glycerol concentration in the dialysate from SCAT increased during exercise (4.3 ± 0.5-fold vs. basal; *p* = 0.002). There was no difference in dialysate glycerol response after oral administration of metformin in Protocol 1 when compared with the glycerol response in the control (i.e., probe perfused with Ringer) in Protocol 2. In Protocol 2, there was no difference in the glycerol response in the probe with locally perfused metformin when compared with the response in control probe perfused with Ringer (**Figure 3B**). Thus, neither type of metformin administration had inhibitory effect on lipolysis induced in SCAT by a single bout of exercise. Similarly, the oral administration of metformin had no effect on circulating glycerol (*p* = 0.46) and fatty acid levels in plasma (*p* = 0.18) (**Figures 3D,E**) at the end of exercise in 210 min.

The lactate levels increased in plasma and in dialysate from SCAT after 30–60 min of exercise (3.6-fold vs. basal; *p* = 0.015; 2.75-fold vs. basal; *p* = 0.002; respectively) (**Figures 3C,F**). Metformin administration did not affect lactate concentration in dialysate (**Figure 3C**) or in plasma (**Figure 3F**).

## Discussion

In this study we have demonstrated that metformin is distributed in SCAT after single oral dose administration, and that metformin administration exerts no inhibitory effect on exercise-induced lipolysis in healthy lean men.

Pharmaco-kinetics measurements have shown that maximal metformin plasma concentrations are typically reached after 2.64 ± 0.82 h (FDA) after orally administered dose. Similarly, in the current study we observed maximal concentrations between 180 and 210 min after the oral administration in SCAT and in plasma. Mean dialysate concentrations were approximately 30% of those in plasma. According to *in vitro* metformin recovery (80%) the concentration in SCAT interstitium might be estimated as 0.55 – 1.68 μg/mL. Nevertheless, the recovery of the probe *in vivo* may differ from that *in vitro* as we have shown before (Siklova-Vitkova et al., 2009), thus these values have to be considered with caution. The limitation of the present study is that it was not possible to measure precise *in vivo* recovery (i.e., concentration) of metformin in SCAT in our subjects, as the “stable-in time” concentration of metformin in SCAT is a necessary condition for the zero-flow method.

It was shown previously, in human and rodent isolated adipocytes, that metformin treatment inhibited catecholamine and ANP-stimulated lipolysis through its action on AMPK (Zhang et al., 2009; Bourron et al., 2010). Metformin activation of AMPK led to the inhibition of phosphorylation of HSL preventing HSL translocation to lipid droplet (Bourron et al., 2010). However, the inhibition of stimulated lipolysis during exercise might be regarded as unfavorable process as fatty acids released by adipose tissue serve as energy substrate for other organs, especially for working muscle.

Importantly, no inhibition of exercise-stimulated lipolysis in SCAT by *in situ* or oral metformin administration was found in the present study. It should be emphasized that previously reported studies dealt with *in vitro* systems (isolated adipocytes) (Zhang et al., 2009; Bourron et al., 2010) and employed supraphysiological (2 mM) concentration of metformin, i.e., approximately 200 times higher than that found in adipose tissue in this study. *In vivo* inhibition of lipolysis in SCAT was shown only after local stimulation of the adrenergic pathway in one study (Flechtner-Mors et al., 1999). Our protocol employs physical activity, which represents physiological and more complex trigger of lipolysis. During local administration, we used high concentration of metformin in perfusate, similar as in published study (Flechtner-Mors et al., 1999). It was much higher than during orally given dose (2550 mg – submaximal recommended dose). Nevertheless, on both occasions no antilipolytic effect was detected in SCAT, which shows that the lack of effect is independent on the local concentration of the metformin in AT.

We used the given exercise intensity as it represents an optimal model to increase lipolysis in SCAT in healthy lean men as shown by Moro et al. (2007). The similar intensity was used in our previous studies focused on lipolysis regulation in SCAT during exercise (Stich et al., 2000a,b).

It should be considered, when interpreting this study, that it was performed in young healthy lean men, while the study Bourron et al. (2010) was carried out in normal to moderately overweight women and the study of Flechtner-Mors et al. (1999) in severely obese women. The impact of metformin on exercise-induced lipolysis in diabetic patients would be warranted in future studies. Furthermore, taken into account that exercise-induced lipolysis is gender-specific (Arner et al., 1990; Moro et al., 2007), it may not be excluded that metformin effect during exercise would be gender-specific, too.

A wide range of regulatory and signaling pathways may play a role in the lipolysis regulation, such as cytokines/myokines IL-6, IL-15 (Ajuwon and Spurlock, 2004), insulin, or FGF21 (Hotta et al., 2009). Indeed, metformin may interfere with these pathways, as it is able to inhibit IL-6 and insulin signaling (Kisfalvi et al., 2009; Li et al., 2014), or FGF21 expression (Kim et al., 2013). Also the role of AMPK in exercise stimulated lipolysis remains controversial (Gaidhu and Ceddia, 2011). Thus, the detailed effects of metformin on AMPK and/or other signaling pathways during physiological conditions, such as physical activity, needs to be investigated in future studies.

It was reported that metformin administration may increase lactate production in diabetic subjects, which may lead to the development of lactate acidosis (Wills et al., 2010). The SCAT is one of the sources of lactate production (Jansson et al., 1990), therefore we analyzed its levels in dialysate and in plasma during exercise. The increase of lactate levels in plasma was observed after 30 min and in SCAT after 60 min of exercise, which suggests that the exercise work-loads applied in this study were close to the individual anaerobic thresholds. However, there was no effect of orally or locally administered metformin on evolution of lactate levels in plasma or in SCAT. This is in line with previous reports, which observed that metformin did not alter circulating lactate concentration during acute bout of exercise (Gudat et al., 1997). But it is difficult to generalize from previous studies as variable results were reported including increased (Hansen et al., 2015) or reduced lactate response to exercise after metformin administration (Johnson et al., 2008).

## Conclusion

We have demonstrated that metformin is distributed in adipose tissue and it shows a similar time-course to plasma following single oral administration. Metformin did not inhibit physiologically increased lipolysis induced by a single bout of exercise in healthy lean men. This suggests that metformin does not play detrimental role in mobilization of lipid energy substrates during exercise.

## Author Contributions

EK performed the experiments, researched the data, and wrote the manuscript. PT performed the experiments, researched the data, and edited the manuscript. IdG performed the experiments. VŠ designed the study, and reviewed and edited the manuscript. MŠ designed the study, researched the data, and wrote the manuscript. EK is the guarantor of this work and, as such, had full access to all the data in the study and takes responsibility for the integrity of the data and the accuracy of the data analysis.

## Conflict of Interest Statement

The authors declare that the research was conducted in the absence of any commercial or financial relationships that could be construed as a potential conflict of interest.
